# Serial computed tomography findings of Coronavirus disease 2019 (COVID-19) pneumonia treated with favipiravir and steroid therapy: report of 11 cases

**DOI:** 10.1186/s42269-021-00553-7

**Published:** 2021-05-19

**Authors:** Naoki Irizato, Hiroshi Matsuura, Atsuya Okada, Ken Ueda, Hitoshi Yamamura

**Affiliations:** 1 Osaka Prefectural Nakakawachi Emergency and Critical Care Center, 3-4-13 Nishiiwata, Higashiosaka, Osaka 578-0947 Japan; 2 Osaka Rosai Hospital, 1189-3 Nagasonecho, Kitaku, Sakai, Osaka 591-8025 Japan

**Keywords:** COVID-19, Steroid, Favipiravir, Ground-glass opacities, Computed tomography, Manifestations

## Abstract

**Background:**

This study evaluated the time course of computed tomography (CT) findings of patients with COVID-19 pneumonia who required mechanical ventilation and were treated with favipiravir and steroid therapy.

**Results:**

Eleven patients with severe COVID-19 pneumonia were included. CT findings assessed at the three time points showed that all patients had ground-glass opacities (GGO) and consolidation and mixed pattern at intubation. Consolidation and mixed pattern disappeared in most of the patients whereas GGO persisted in all patients at 1-month follow-up. In addition to GGO, a subpleural line and bronchus distortion and bronchial dilatation were frequent findings. The degree of resolution of GGO varied depending on each patient. The GGO score correlated significantly with the time from symptoms onset to initiation of steroid therapy (*ρ* = 0.707, *p* = 0.015).

**Conclusions:**

At 1-month follow-up after discharge, non-GGO lesions were absorbed almost completely, and GGO were a predominant CT manifestation. Starting steroid therapy earlier after onset of symptoms in severe COVID-19 pneumonia may reduce the extent of GGO at 1-month follow-up.

## Background

The spread of severe acute respiratory syndrome coronavirus 2 throughout the world has created the pandemic of coronavirus disease 2019 (COVID-19). COVID-19 is essentially diagnosed by real-time reverse transcriptase-polymerase chain reaction (PCR) testing, and chest high-resolution computed tomography (CT) is also central to the diagnosis of COVID-19 pneumonia (Lv et al. 2020). COVID-19 pneumonia is sometimes progressively presented as acute respiratory distress syndrome (ARDS) and is a definite factor of severe disease status (Marini and Gattinoni 2020). The features of COVID-19 pneumonia are often limited and are usually characterized initially by a pattern of ground-glass opacities (GGO) on CT that signifies interstitial edema rather than alveolar edema (Salehi et al. 2020).

To reduce the viral genome amounts, COVID-19 needs to be treated with a complex strategy that includes anti-inflammatory drugs and anticoagulants. To date, several drugs have been used to reduce viral genome amounts, such as chloroquine, lopinavir/ritonavir, remdesivir, and favipiravir (Wiersinga et al. 2020). Favipiravir selectively and potently inhibits the RNA polymerase of RNA viruses and is expected to be a primary drug in COVID-19 treatment (Furuta et al. 2017).

The use of favipiravir, steroid, and heparin has been attempted to treat COVID-19 (Yamamura et al. 2020). This treatment strategy for COVID-19-associated ARDS may bring beneficial results, especially in terms of prognosis and CT manifestations. The serial findings of CT manifestations of COVID-19-associated ARDS remain unclear.

This study investigated the progression of CT manifestations of severe COVID-19-associated ARDS treated with favipiravir and steroid therapy.

## Methods

### Patients

We retrospectively reviewed the records of 11 patients admitted to our hospital after being transferred from other hospitals and who required mechanical ventilation for severe COVID-19 pneumonia from April 2 to 27, 2020. COVID-19 pneumonia was confirmed by real-time reverse transcriptase-PCR of specimens obtained from nasopharyngeal and throat swabs performed in approved laboratories and by chest CT. Inclusion criteria were patients with no history of other pulmonary infectious disease who required mechanical ventilation for respiratory failure due to COVID-19 pneumonia. The patients underwent CT examinations in our department at intubation (on admission), at discharge, and at 1-month follow-up. We also examined the patients’ laboratory data, including C-reactive protein and lactate dehydrogenase levels. The Ethical Committee of the hospital approved this study (approval no.: 02–0546-A).

### Treatment protocol

We administered favipiravir, a steroid, and heparin to treat COVID-19 according to the following treatment protocol: oral favipiravir at 3600 mg (day 1) and 1600 mg (days 2 to 14), methylprednisolone at 1000 mg for 3 days (begun on day 5 from initial favipiravir administration (Ojha et al. 2020)), and low molecular weight heparin at 2000 IU every 12 h or unfractionated heparin at 10,000–12,000 IU/day. Heparin was administered following intubation and the initiation of mechanical ventilation.

### CT scanning protocol

All patients underwent scanning with an IQon Spectral CT system (Philips Healthcare). The following scanning protocol was used: supine patient position; 120-kV tube voltage; 162–265 mAs tube current-exposure time product; 0.891 pitch; 64 × 0.625-mm collimation; 0.27-s rotation time; and 1- and 5-mm section thicknesses after reconstruction. All CT scans were unenhanced.

### Imaging interpretation

Two experienced radiologists (12 and 27 years of experience) blindly and independently reviewed the chest CT images. They viewed all images with both lung (width, 1600 Hounsfield Units [HU]; level, -600 HU) and mediastinal (width, 360 HU; level, 60 HU) settings.

Chest CT scanning was regularly performed at three different time points: at intubation (transfer from another hospital due to respiratory worsening), discharge, and 1-month follow-up after discharge.

CT findings were classified into two major categories (Ojha et al. 2020): major patterns of increased attenuation and ancillary findings. Major patterns of increased attenuation were classified as follows: GGO, consolidation, mixed pattern (GGO + consolidation), and reticular pattern. Ancillary findings were classified as follows: interlobular septal thickening, intralobular septal thickening, crazy-paving, vascular enlargement, reversed halo sign, peri-lobular pattern, air bronchogram, bronchial wall thickening, subpleural line, nodule, pleural effusion, pleural thickening, lymphadenopathy, pericardial effusion, cavitation, pulmonary fibrosis, bronchus distortion, bronchial dilatation, and atelectasis.

To quantify the extent of major patterns of increased attenuation at 1-month follow-up after discharge, CT scores were assessed based on the area involved. Bilateral lungs were divided into 6 lung zones: right upper, middle, and lower lobes, left upper lobe other than the lingula region, left lung lingula, and left lower lobe. After zoning, the percentage of involvement was scored for each lung zone as follows: score 0, no involvement; 1, < 10% involvement; 2, 10% to < 30% involvement; 3, 30% to < 50% involvement; and 4, ≥ 50% involvement. The recorded scores of each lung zone were summed, and the maximum score was 24. Any disagreements remaining after separate evaluations of CT findings and scoring were performed were resolved through discussion and consensus.

### Statistical analysis

Patient age is shown as the mean ± SD, and all categorical variables are shown as counts and percentages. The Student *t*-test was used to compare the radiologic scores based on CT findings between two groups (the period from onset to steroid use before 14 days versus that after 14 days). Statistical analyses were conducted with JMP Version 13.0.0 (SAS Institute Inc.).

## Results

### Patient characteristics

The patients’ clinical characteristics and laboratory data are listed in Table 1. Nine patients were men. A history of hypertension was present in 7, diabetes in 5, and sleep apnea syndrome in 2. All patients required mechanical ventilator support from admission. The mean time from first symptom appearance to intubation was 10.6 days. The mean PaO_2_/FIO_2_ ratio and mean PEEP at intubation were 210 and 10 cmH_2_O, respectively. The median number of ventilator days was 10.1 days. The mean time from the appearance of the first symptom to the administration of favipiravir was 9.0 days, heparin was 11.5 days, and steroid was 13.7 days. Chest CT scanning was performed 3 times: at intubation, at discharge, and at 1 month after discharge, with the median time from first symptom appearance to each time point being 10.3 days, 21.5 days, and 55.5 days, respectively.

**Table 1:** Characteristics of the 11 patients with COVID-19

*N* = *11*	Total
Age, mean (SD), years	61 (12)
Male sex, No. (%)	9 (82)
Body weight, mean (SD), kg	71 (23)
Diabetes mellitus, No. (%)	5 (45)
Hypertension, No. (%)	7 (63)
Sleep apnea syndrome, No. (%)	2 (18)
PaO_2_/FIO_2_ ratio at intubation, mean (SD)	210 (82)
PEEP at intubation, mean (SD), cmH_2_O	10 [10, 12]
LDH on admission, median [IQR], IU/L	504 [387, 582]
CRP on admission, median [IQR], mg/dL	12.7 [4.8, 19.1]
PCT on admission, median [IQR], ng/mL	0.19 [0.1, 0.36]
Time from first symptom appearance, mean (SD), days	
At intubation	10.3 (2.9)
At discharge	21.5 (2.9)
1 month after	55.5 (9.8)
Time from first symptom appearance, mean (SD), days	
Favipiravir	9.0 (2.3)
Heparin	11.5 (3.4)
Steroid	13.7 (2.7)
Intubation	10.6 (2.8)
Symptoms at 1 month, No (%)	
Muscle weakness	8 (73)
Fatigability	6 (55)
Body pain	3 (27)
Dyspnea	2 (18)
Cough	0 (0)
Ventilator days, median [IQR], days	10.1 (2.2)

SD, standard deviation; IQR, interquartile range; PEEP, positive end-expiratory pressure; LDH, lactate dehydrogenase; CRP, C-reactive protein; PCT, procalcitonin

### CT findings

CT findings in the 11 patients at the three time points are shown in Table 2. All 11 patients had GGO and consolidation and mixed pattern at intubation, and the findings showed the percentage of incidence of a particular finding at the three fixed time points of follow-up CT scans.

**Table 2:** CT findings of the 11 patients at three time points

CT findings	At intubation	At discharge	1-M follow-up
Major patterns of increased attenuation			
GGO	100	100	100
Consolidation	100	81.8	9.1
GGO + Consolidation (mixed pattern)	100	72.7	9.1
Reticular pattern	0	4.5	0
Ancillary findings			
Interlobular septal thickening	63.6	9.1	0
Intralobular septal thickening	81.8	9.1	0
Crazy-paving	18.2	0	0
Vascular enlargement	0	0	0
Reversed halo sign	0	0	0
Peri-lobular pattern	18.2	0	0
Air bronchogram	90.9	18.2	0
Bronchial wall thickening	9.1	18.2	4.5
Subpleural line	4.5	81.8	68.2
Nodule	13.6	27.3	27.3
Pleural effusion	45.5	54.5	0
Pleural thickening	0	0	0
Lymphadenopathy	0	0	0
Pericardial effusion	0	0	0
Cavitation	0	0	0
Pulmonary fibrosis	0	4.5	4.5
Bronchus distortion	45.5	36.4	36.4
Bronchial dilatation	36.4	27.3	27.3
Atelectasis	77.3	22.7	0

Values are percentages

CT, computed tomography; GGO, ground-glass opacities

**Fig. 1:**
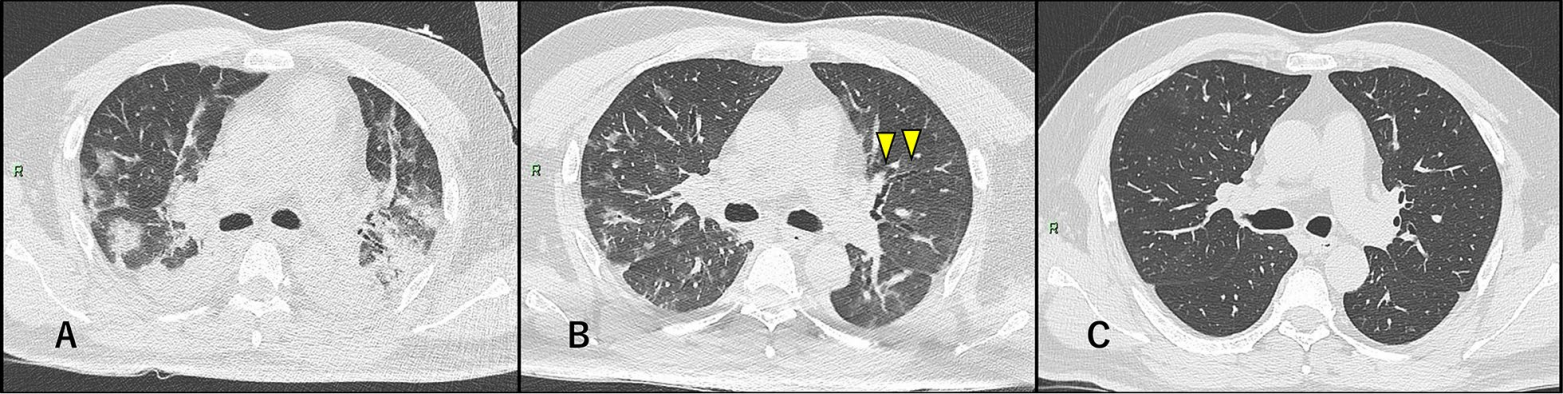
Severe COVID-19 pneumonia in a 50-year-old man. **a** At intubation (10 days after symptoms onset): multiple GGO and bilateral, peripheral, and posterior consolidations were observed. **b** At discharge (22 days after symptoms onset). The consolidation lesions had converted to GGO and remained as diffuse GGO with lower density than in (**a**). Bronchus distortion and bronchial dilatation were also observed. **c** At 1-month follow-up (50 days after symptoms onset): Most of abnormalities had obviously been absorbed

**Fig. 2:**
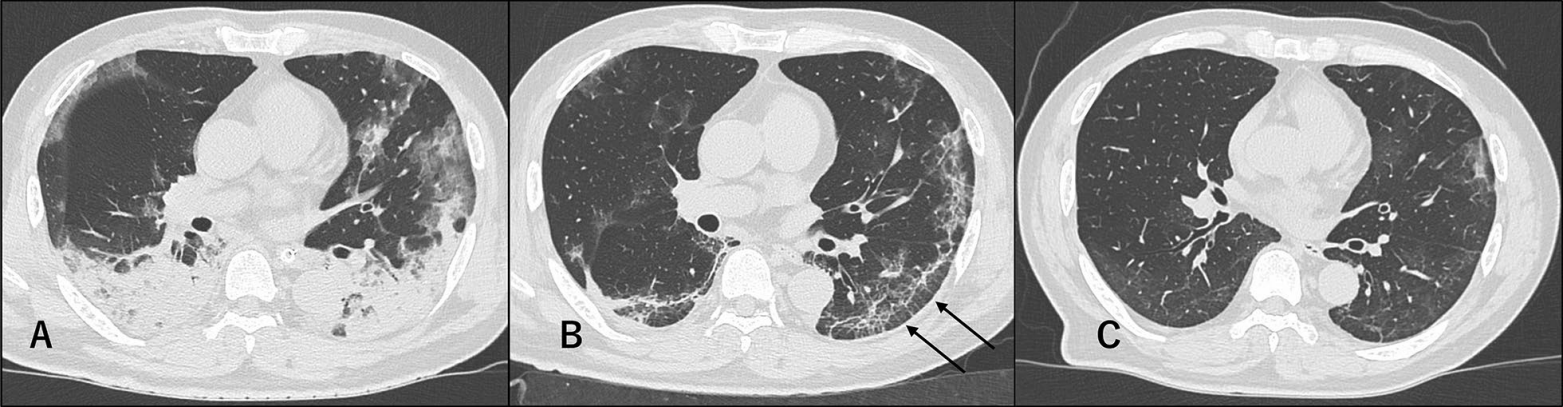
Severe COVID-19 pneumonia in a 59-year-old man. **a** At intubation (14 days after symptoms onset): Multiple GGOs and consolidation in the subpleural areas of both lungs were observed. Air bronchogram was also detected inside the consolidation. **b** At discharge (21 days after symptoms onset): Bilateral and posterior subpleural lines and interlobular septal thickening (arrows) appeared. **c** At 1-month follow-up (53 days after symptoms onset). GGOs with decreased extent and density could still be observed

Although consolidation (81.8%) and mixed pattern (72.7%) were still present in most patients at discharge, the regions occupied and their density had decreased with improvement of the patients’ respiratory condition, as shown in a previous report (Yamamura et al. 2020), and some areas of consolidation had converted to GGO. At the 1-month follow-up, consolidation and mixed pattern had disappeared in most of the patients, but GGO were persistent in all 11 patients. The degree of resolution of GGO varied depending on each patient. Some showed significant resolution, whereas others showed huge extents of GGO remaining at this time (Figs. 1, 2).

**Fig. 3:**
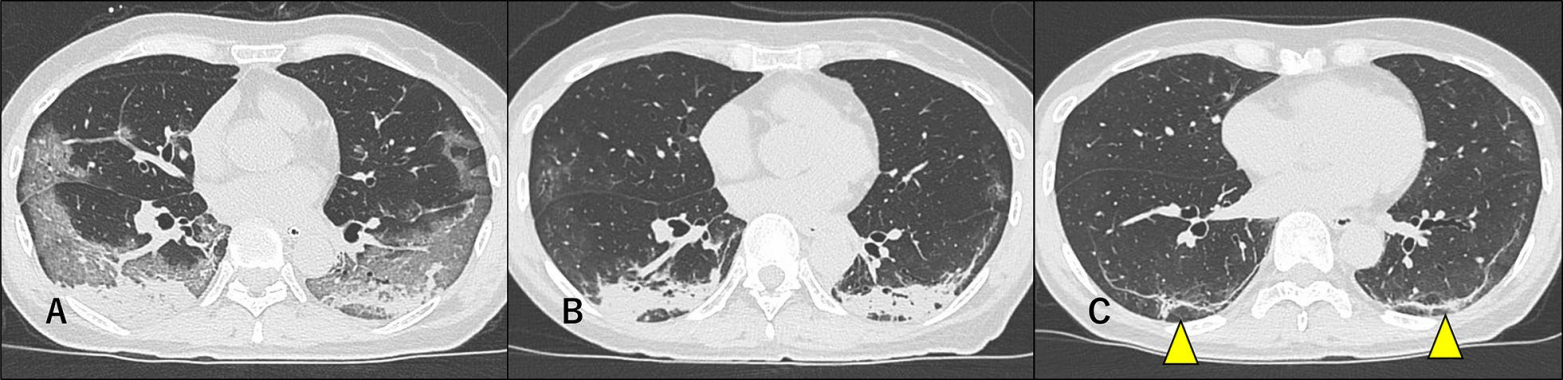
Severe COVID-19 pneumonia in a 62-year-old woman. **a** At intubation (8 days after symptoms onset): Bilateral, subpleural, and non-segmental GGOs and posterior consolidation were observed. **b** At discharge (18 days after symptoms onset): Bilateral and posterior subpleural lines appeared. **c** At 1-month follow-up (47 days after symptoms onset): The subpleural lines were still present (arrowheads)

**Fig. 4:**
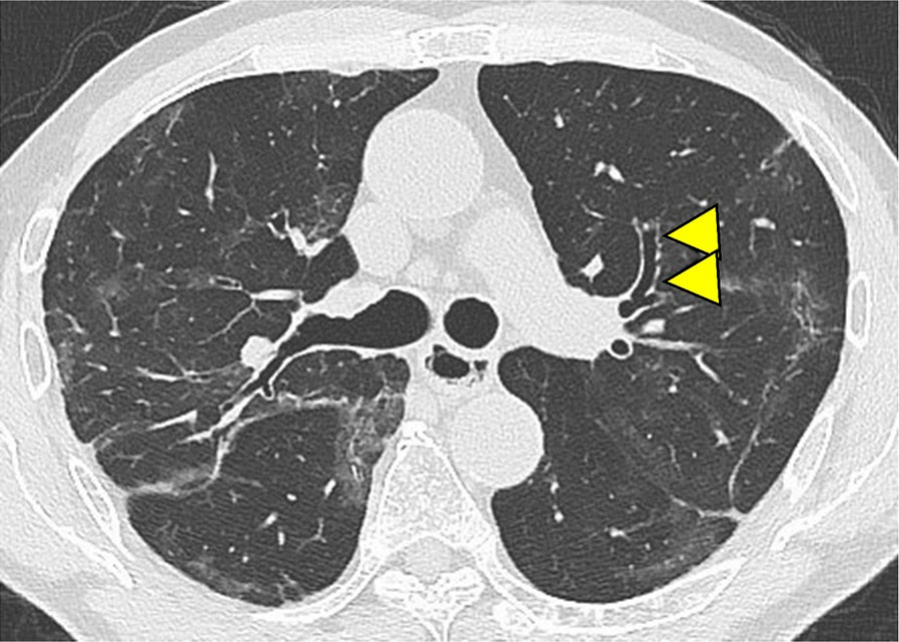
Severe COVID-19 pneumonia in a 79-year-old man at 1-month follow-up after discharge (59 days after symptoms onset). Bronchus distortion and bronchial dilatation were observed (arrowheads). Multiple GGOs remained in both lungs

Among the ancillary findings, most patients had air bronchogram (90.9%), atelectasis (77.3%), intralobular septal thickening (81.8%), and interlobular septal thickening (63.6%) at intubation. Pleural effusion (45.5%), bronchus distortion (45.5%), and bronchial dilatation (36.4%) were detected in some patients. The frequencies of interlobular septal thickening (9.1%), intralobular septal thickening (9.1%), air bronchogram (18.2%), and atelectasis (22.7%) were significantly decreased at discharge, whereas the frequency of subpleural lines increased and became the most frequent ancillary finding at discharge (81.1%). Subpleural lines were mainly detected in the posterior area of the lungs where consolidation had disappeared. Many patients showed these findings even at the 1-month follow-up (68.2%) (Fig. 3). Bronchus distortion and bronchial dilatation were detected in about 30–40% of the patients at every time point (Fig. 4).

### CT score at 1-month follow-up

**Fig. 5:**
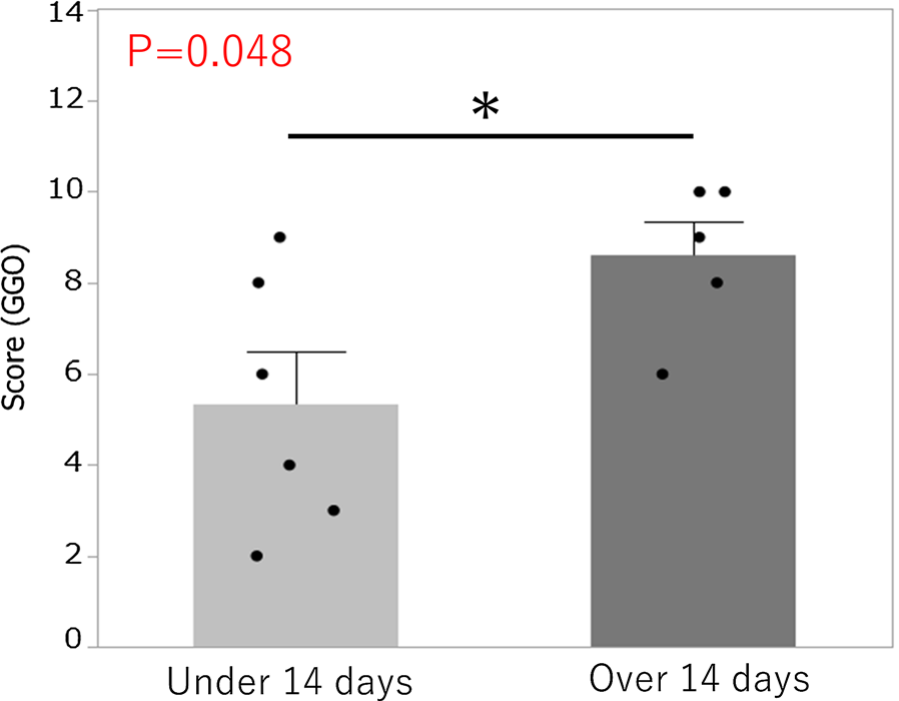
CT score at 1-month follow-up. The GGO scores of the patients receiving steroid therapy after 14 days were significantly higher than those of the patients who received steroid therapy before 14 days (score: 8.6 vs 5.3, *p* = 0.048)

The GGO score and all findings at the 1-month follow-up were calculated based on CT findings. The GGO scores of the 6 patients who were administered steroid therapy after 14 days from the onset of initial symptoms were significantly higher than those of the 5 patients receiving steroid before 14 days (score: 8.6 vs 5.3, *p* = 0.048) (Fig. 5). There were no other correlations between any radiologic features and any lab markers or clinical data including 1-month follow up, but most of the patients had residual symptoms such as general fatigue.

## Discussion

This study reported the serial CT findings of COVID-19 pneumonia treated with favipiravir and an anti-inflammatory strategy. We found that non-GGO lesions, such as consolidation, mixed pattern, and reticular pattern, showed a faster rate of resolution than GGO in the convalescence period, and GGO remained at the 1-month follow-up in all patients although their extent and density had decreased.

Early in COVID-19 pneumonia, GGO appear in a single or a few regions as peripheral lesions. However, the advanced phase of COVID-19 pneumonia is associated with significantly increased GGO plus mixed GGO and consolidation in the bilateral dorsal regions and also shows specific findings such as a reticular pattern (Shi et al. 2020; Zhao et al. 2020). The advanced phase or peak stage was reported to be 9–13 days after the onset of initial symptoms, and the CT findings including diffuse GGO, consolidation, and residual parenchymal bands become most prevalent at this time (Pan et al. 2020). The period after 14 days from the onset of initial symptoms is called the absorption stage, during which the infection is under control and the lesions are gradually being absorbed (Pan et al. 2020). The serial changes in CT imaging show similarities with the time course of clinical severity (Huang et al. 2020). All patients in this study were transferred from other hospitals due to hypoxia and dyspnea and required mechanical ventilation. Thus, the first CT imaging was performed at intubation in this study, and the median number of days from onset of symptoms was 10.5 (7.25–13) days, during which findings typical of the peak stage might be observed. The initial CT findings in our study showed bilateral peripheral and central GGO, consolidation, and mixed pattern in almost all patients, and the CT features were similar to those reported in previous studies (Shi et al. 2020; Zhao et al. 2020).

Although some previous studies reported COVID-19 CT features, studies of serial changes of CT imaging in severe COVID-19 pneumonia over more than 1 month are still inadequate. In a previous study, the overall percentage of complete radiological resolution in patients with mildly severe COVID-19 pneumonia was 53% at 3 weeks after discharge (Liu et al. 2020), and our results showed that severe COVID-19 pneumonia might require a longer time than that. Some patients showed complete radiological resolution in this study (Fig. 1), and thus, further study and follow-up are necessary to clarify this issue.

Severe acute respiratory syndrome coronavirus (SARS-CoV) was a serious infectious disease that occurred in different areas of the world in 2002. CT imaging at 3 months after onset showed GGO, reticular pattern, and consolidation in every patient at follow-up, and GGO and reticular pattern still remained in 90% of the patients even at 84 months of follow-up (Wu et al. 2016). In the present study, the reticular pattern and consolidation seen on CT imaging of severe COVID-19 pneumonia disappeared quickly compared to that in the 2002 SARS-CoV, and GGO became predominant at 1-month follow-up. The mortality and severity of COVID-19 pneumonia are considered low (Wang et al. 2020), and our CT findings in the subacute to chronic phase showed milder symptoms compared to those of SARS-CoV in 2002. In patients with ARDS, GGO (57.9%) and airway disease (50%) remained on CT follow-up 6 months later (Masclans et al. 2011), and GGO were still detected in 42% of patients even at 5 years after severe ARDS (Wilcox et al. 2013). Continued follow-up and comparison of the reversibility of residual CT findings in severe COVID-19 pneumonia might help in understanding the pathophysiological differences between severe COVID-19 pneumonia and other related pneumonias.

Limitations of this study include the small sample size and data collection only from a single center. Second, our CT score does not reflect the degree of density of GGO. GGO on CT imaging differ not only in volume but also in HU readings of the GGO area. Third, the relationship between GGO score and clinical data such as respiratory function was not assessed. Finally, as this study is an observational study, there may be unknown confounding factors. We expect that the present findings will help in understanding the pathophysiological mechanism of severe COVID-19 pneumonia in the future.

## Conclusions

In 11 patients with severe COVID-19 pneumonia, non-GGO lesions were absorbed almost completely, and GGO were a predominant CT manifestation at 1-month follow-up after discharge. Further follow-up is needed to evaluate the reversibility of the radiological abnormalities of these patients with severe COVID-19 pneumonia.

## Data Availability

The datasets generated for this study are available on request to the corresponding author.

## Abbreviations

COVID-19: Coronavirus disease 2019
CRP: C-reactive protein
IQR: Interquartile range
GGO: Ground-glass opacities
LDH: Lactate dehydrogenase
PCR: Polymerase chain reaction
PCT: Pro-calcitonin
PEEP: Positive end-expiratory pressure
SARS-CoV: Severe acute respiratory syndrome coronavirus
SD: Standard deviation
